# Exercise endurance capacity is markedly reduced due to impaired energy homeostasis during prolonged fasting in FABP4/5 deficient mice

**DOI:** 10.1186/s12899-019-0038-6

**Published:** 2019-03-13

**Authors:** Tatsuya Iso, Hikari Haruyama, Hiroaki Sunaga, Miki Matsui, Hiroki Matsui, Rina Tanaka, Yogi Umbarawan, Mas Rizky A. A. Syamsunarno, Tomoyuki Yokoyama, Masahiko Kurabayashi

**Affiliations:** 1 Department of Cardiovascular Medicine, Gunma University Graduate School of Medicine, 3-39-22 Showa-machi, Maebashi, Gunma 371-8511 Japan; 2 Department of Laboratory Sciences, Gunma University Graduate School of Health Sciences, 3-39-22 Showa-machi, Maebashi, Gunma 371-8511 Japan; 3 Department of Internal Medicine, Faculty of Medicine Universitas Indonesia, Jl. Salemba Raya no. 6, Jakarta, 10430 Indonesia; 4 Department of Biochemistry and Molecular Biology, Universitas Padjadjaran, Jl. Raya Bandung Sumedang KM 21, Jatinangor, West Java 45363 Indonesia

**Keywords:** Metabolism, Exercise, Fasting, Fatty acid, Glucose, Skeletal muscle, Liver, FABP4, FABP5

## Abstract

**Background:**

Skeletal muscle prefers carbohydrate use to fatty acid (FA) use as exercise intensity increases. In contrast, skeletal muscle minimizes glucose use and relies more on FA during fasting. In mice deficient for FABP4 and FABP5 (double knockout (DKO) mice), FA utilization by red skeletal muscle and the heart is markedly reduced by the impairment of trans-endothelial FA transport, with an increase in glucose use to compensate for reduced FA uptake even during fasting. We attempted to determine whether prolonged fasting affects exercise performance in DKO mice, where constant glucose utilization occurs.

**Results:**

A single bout of treadmill exercise was performed in the fed and fasted states. The initial speed was 10 m/min, and gradually increased by 5 m/min every 5 min up to 30 m/min until the mice stopped running. Running distance was significantly reduced by DKO genotype and prior fasting, leading to the shortest distance in fasted DKO mice. Levels of glycogen in skeletal muscle and the liver were nearly depleted in both WT and DKO mice during prolonged fasting prior to exercise. Levels of TG in skeletal muscle were not reduced by exercise in fasted DKO mice, suggesting that intramuscular TG was not utilized during exercise. Hypoglycaemia was accelerated in fasted DKO mice, and this acceleration could be due to constant glucose utilization by red skeletal muscle and the heart where FA uptake is diminished due to defective trans-endothelial FA transport. Taken together, energy supply from serum and storage in skeletal muscle were very low in fasted DKO mice, which could lead to a significant reduction in exercise performance.

**Conclusions:**

FABP4/5 have crucial roles in nutrient homeostasis during prolonged fasting for maintaining exercise endurance capacity.

## Background

Fatty acid binding proteins (FABPs) are 14–15 kDa cytosolic proteins that can reversibly bind to hydrophobic molecules such as saturated and unsaturated fatty acids (FAs) and eicosanoids with high affinity [1, 2]. It has been reported that FABPs promote the transport of lipids to specific compartments in the cells. Among FABPs, FABP4 (also referred to as A-FABP/aP2/ALBP) and FABP5 (also known as E-FABP/mal1) are abundantly expressed in adipocytes, macrophages and muscle-type capillary endothelial cells [1–5]. A series of studies with mice lacking both FABP4 and FABP5 (double knockout (DKO) mice) provided evidence that FABP4/5 are involved in the development of metabolic diseases, including diet-induced obesity, type 2 diabetes and insulin resistance [6–8]. Although it has been reported that both local and systemic inflammatory processes mediated by adipocyte and macrophage FABP4/5 can cause such metabolic diseases [1, 2, 6, 7, 9, 10], it remains obscure how the simultaneous disruption of FABP4/5 in mice exhibits remarkable effects on the amelioration of metabolic diseases.

Recent studies from our laboratory and others have reported that capillary endothelial FABP4/5 have an important role in FA transport via circulation into the heart and skeletal muscle (referred to as trans-endothelial FA transport) [3, 11–13]. A tracing study using ^125^I-BMIPP (FA tracer) and ^18^F-FDG (glucose tracer) revealed that DKO mice exhibit reduced FA uptake with a marked increase in glucose use in red/oxidative skeletal muscle and the heart even during fasting. Thus, trans-endothelial FA transport occurs in highly oxidative tissues with muscle type continuous capillaries, while a remarkable increase in glucose use is induced in the same tissues by a compensatory mechanism independently of insulin. These findings suggest that the ameliorating effects of FABP4/5 disruption observed in diseased models could be due to a compensatory increase in glucose use by the peripheral tissues, at least in part.

In contrast to the beneficial effects on metabolic diseases, DKO mice also exhibit an impairment of systemic metabolism when fasting is prolonged. In the fasted state in general, most tissues, except red blood cells and the brain, rely on FA use to produce energy and minimize glucose consumption [14, 15]. When fasting is prolonged, the hydrolysis of triacylglycerol (TG) in adipose tissue is accelerated, leading to an increase in plasma levels of non-esterified FA (NEFA) as the major energy substrate [14, 15]. Glycogen content in the liver becomes nearly depleted after a 24-h fast, while gluconeogenesis is accelerated to supply glucose to glucose-dependent peripheral tissues and cells [14, 15]. On the other hand, in DKO mice in the fasted state, plasma levels of NEFA are more elevated due to reduced FA uptake, which results in hepatosteatosis and hyperketonaemia [16]. In addition, the compensatory use of glucose in red skeletal muscle and the heart leads to severe hypoglycaemia, which is also at least partly caused by a reduction in gluconeogenesis [16]. Moreover, thermogenesis severely declines in fasted DKO mice when they are exposed to cold environment [17]. Major energy substrates for thermogenesis, such as glycogen in skeletal muscle, TG in brown adipose tissue and plasma glucose, become less available because they are depleted rapidly in DKO mice, which results in fatal hypothermia [17]. Thus, in DKO mice, metabolic adaptation to prolonged fasting is severely impaired due to defective FA utilization by red skeletal muscle and the heart, a compensatory use of glucose and the deranged redistribution of energy storage.

Exercise intensity influences the preference for energy substrates by skeletal muscle. When exercise intensity is low, the primary fuels are NEFA, released from adipose tissue via lipolysis, and glucose, derived from oral ingestion or the liver (gluconeogenesis or glycogenolysis) [18, 19]. As exercise intensity increases, the use of plasma glucose progressively increases, whereas the utilization of circulating NEFA declines. Simultaneously, the contribution of muscle glycogen progressively increases as well. When moderate intensity exercise continues, the contribution of the lipid oxidation in skeletal muscle is enhanced [18, 19]. Thus, exercise at a high-intensity enhances the relative contribution of carbohydrates, whereas exercise at a low to moderate intensity promotes the combustion of both carbohydrates and lipids as fuels [18, 19].

Metabolic homeostasis is impaired in DKO mice in response to fasting as described above. In this study, therefore, we studied whether exercise endurance is impaired more severely in DKO mice in the fasted state because energy provision to skeletal muscle is likely to be impaired during fasting. We found that in addition to reduced FA uptake, blood glucose levels and intramuscular energy storage were markedly decreased during fasting, which could cause a significant reduction in exercise endurance capacity in DKO mice. Our study suggests that FABP4/5 have crucial roles in nutrient homeostasis during prolonged fasting for maintaining exercise endurance capacity.

## Methods

### Animals and sample preparation

FABP4 and FABP5 double knockout (DKO) mice with the C57BL6j background were generated as described previously [7]. Control male wild-type (WT) mice with the C57BL6j background were purchased from Japan SLC Inc. one week before treadmill exercise training. The age (10 to 12 weeks) and body weight (22 to 27 g) of WT and DKO mice were comparable. The mice were housed and fed as described previously [20]. To reduce pain for euthanasia, the mice were briefly anaesthetized in an isoflurane-filled box to induce early unconsciousness, and then maintained using a mask type isoflurane inhalation system during blood sampling. Blood was collected from the retro-orbital plexus, and then centrifuged at 1500×*g* for 15 min at 4 °C to obtain the serum for measurement of biochemical parameters. After cervical dislocation, samples of the liver and skeletal muscle were dissected, snap frozen in liquid nitrogen and stored at − 80 °C until further use. All study protocols were approved by the Institutional Animal Care and Use Committee (Gunma University Graduate School of Medicine). Animal experiments were performed according to the NIH guidelines (Guide for the Care and Use of Laboratory Animals).

### Treadmill exercise testing

To determine the exercise endurance capacity, treadmill exercise testing was carried out using a 2-lane motorized rodent treadmill (MK-680, Muromachi Kikai, Tokyo, Japan) [20, 21]. Mice were accustomed to the treadmill running twice, and then a single bout of running was carried out as described previously (Fig. 2b) [20, 21]. When mice stopped running more than 3 s on the electric shocker, we defined the time point as exhaustion. The total running distance for each mouse was calculated.

### Measurements of glycogen and triacylglycerol

Liver and skeletal muscle were snap frozen in liquid nitrogen and then powder-pulverized. Glycogen and TG were extracted and then measured as described previously using the glycogen assay kit (BioVision, CA) and the Triglyceride E-test Wako (Wako Chemical, Osaka), respectively [3, 16].

### Quantitative analysis of mRNA expression

Total RNA was isolated from liver and skeletal muscle and quantitative real time-PCR were carried out as described previously [3, 17]. The expression levels of the target genes were normalized to the level of glyceraldehyde 3-phosphate dehydrogenase (GADPH) mRNA. The Perfect Real Time Primers (ready-to-use gene-specific primers purchased from Takara Bio Inc., Shiga, Japan) were used.

### Determination of myosin heavy chain isoforms in skeletal muscle

Isoform expression of myosin heavy chain (MHC) in the soleus was determined by an immunofluorescence as described previously [22]. Primary antibodies against MHC I (BA-F8) and MHC IIa (SC-71) were purchased from the Developmental Studies Hybridoma Bank (University of Iowa). Secondary antibodies (Alexa Fluor 350 IgG2b for MHC I and Alexa Fluor 555 IgG1 for MHC IIa) were purchased from Invitrogen. Wheat germ agglutinin conjugated with Alexa Fluor 488 (Invitrogen) was used to stain plasma membrane of each muscle fibre. A combination of type IIb and IIx fibres was recognized as muscle fibres negative for MHC I and MHC IIa. Muscle fibres positive or negative for each MHC isoform were counted and presented as percentage of fibre types.

### Data analysis

IBM SPSS (version 24 for Windows, IBM, NY, USA) was used for statistical analysis. The results are shown as the mean ± standard deviation. A two-way analysis of variance (ANOVA) was utilized to determine the effects of feeding status (fed or fasted) on running distance between WT and DKO mice (DKO genotype). A three-way ANOVA was utilized to analyse interaction effects on gene expression and metabolites levels among exercise, DKO genotype or feeding status. *P* < 0.05 was considered significant. *p < 0.05, **p < 0.01, ***p < 0.001.

## Results

### A remarkable reduction in running distance in fasted FABP4/5 DKO mice

**Fig. 1:**
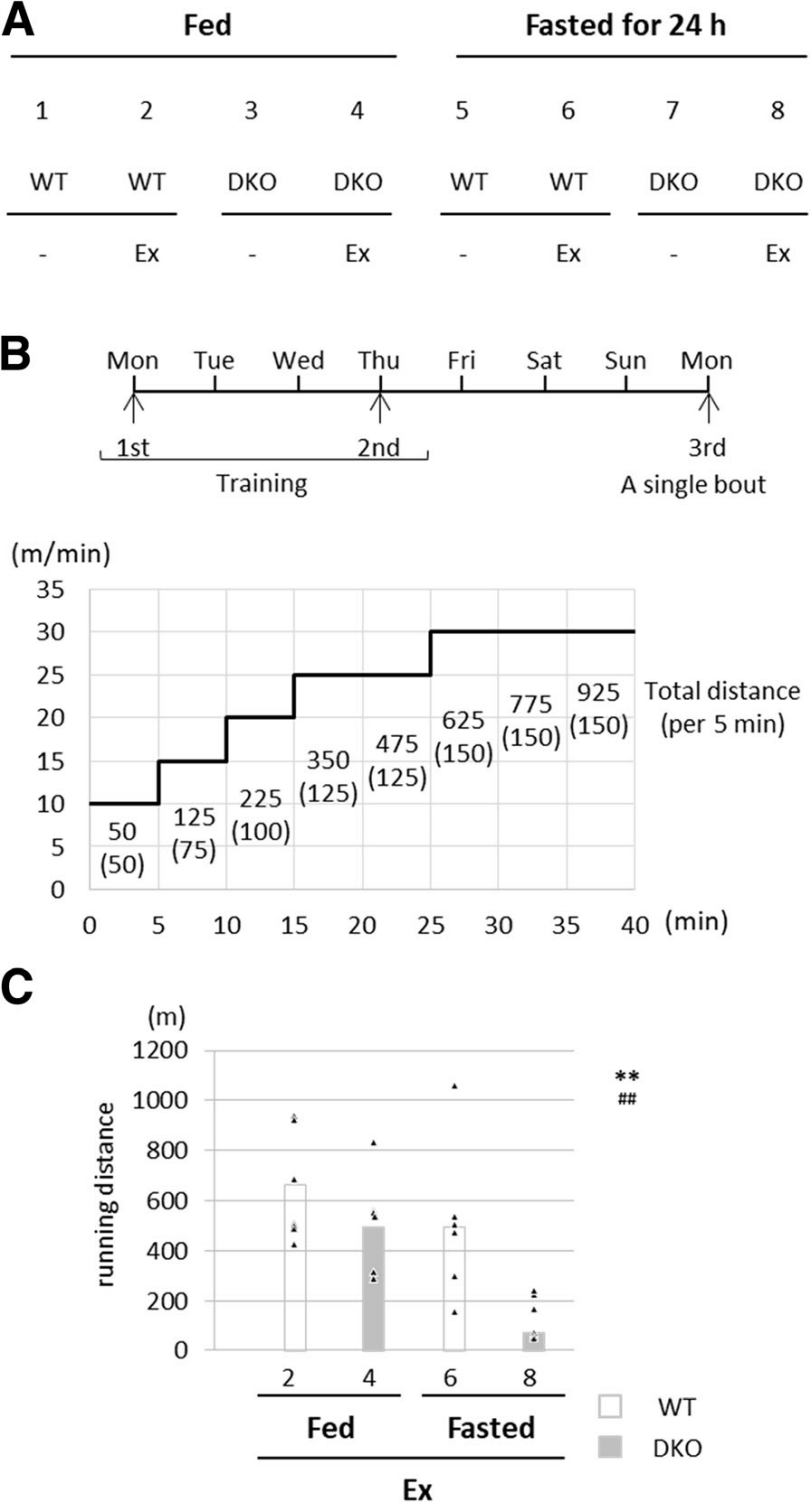
Running distance is significantly decreased in fasted DKO mice. (A) Eight groups prepared for this study. Ex, exercise. (B) The protocol used in this study. See the method in detail. (C) Running distance for each group. *n* = 6. **p < 0.01, main effect for genotype. ^##^*p* < 0.01, main effect for fasting

To test that running endurance is affected by feeding conditions in FABP4/5 DKO mice, a single bout of treadmill exercise was performed in the fed and fasted states (Fig. 1a and b). As shown in Fig. 1c, running distance was markedly reduced by fasting and DKO genotype, leading to the shortest distance in fasted DKO mice.

### Accelerated hypoglycaemia in fasted DKO mice

**Fig. 2:**
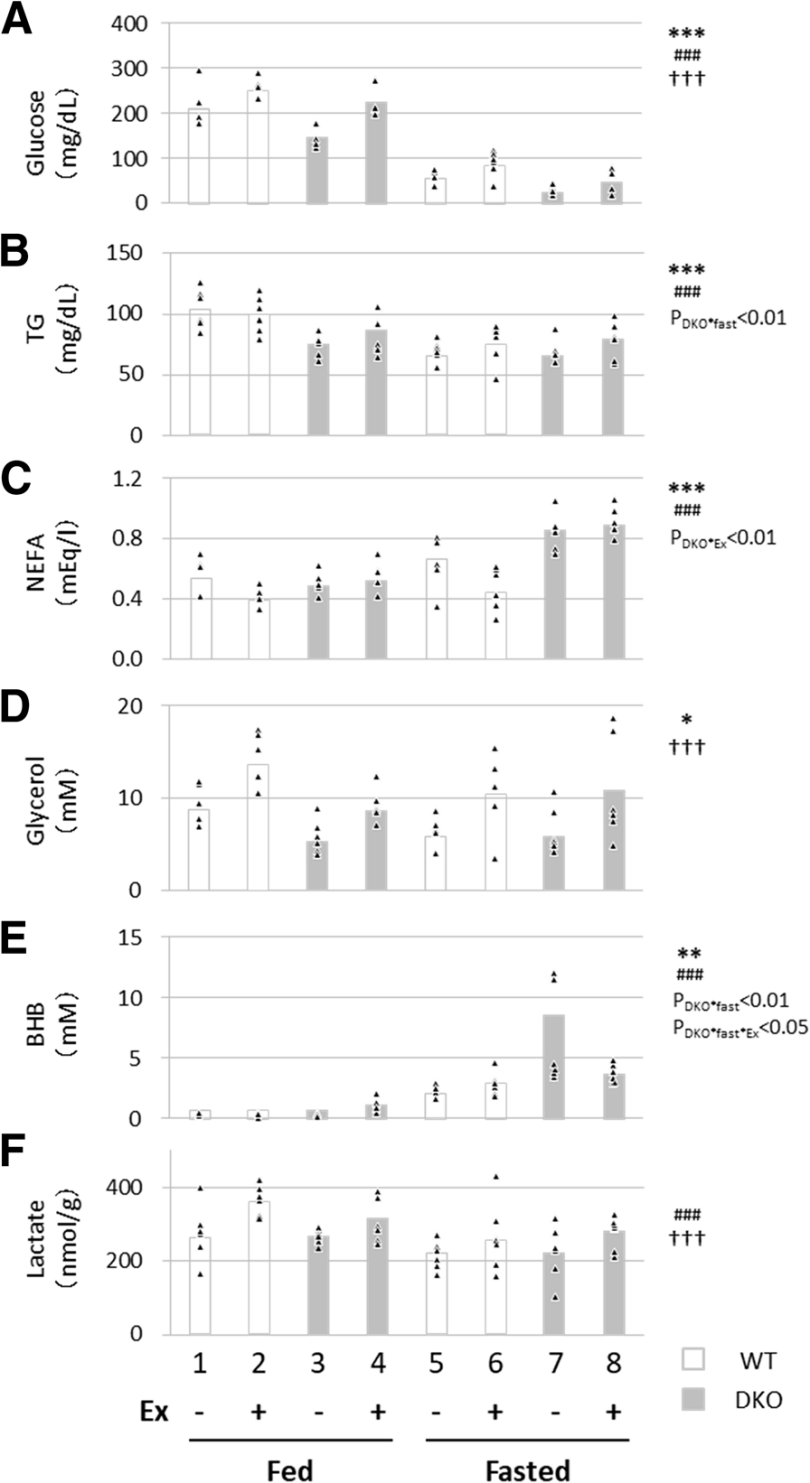
Measurement of metabolites in serum. Blood was collected with or without treadmill exercise to measure serum levels of glucose (A), TG (B), NEFA (C), glycerol (D), BHB (E) and lactate (F) in the fed or fasted state. TG, triacylglycerol; NEFA, non-esterified fatty acid; BHB, β-hydroxybutyrate. *n* = 6. *p < 0.05, **p < 0.01, ***p < 0.001, main effect for genotype. ^###^*p* < 0.001, main effect for fasting. ^**†††**^*p* < 0.001, main effect for exercise. Interaction is indicated with the *p*-value in the figure if it was statistically significant

In search for the reasons for a reduction in endurance capacity in DKO mice during fasting, we measured biochemical parameters in the fed and fasted states with and without treadmill exercise. Serum levels of glucose were reduced by DKO genotype and fasting and increased by exercise, resulting in the lowest level in fasted DKO mice without exercise (Fig. 2a). Serum levels of NEFA were elevated by DKO genotype and fasting (Fig. 2c). Importantly, serum levels of NEFA were reduced by exercise in WT mice, while they were not altered in DKO mice (Fig. 2c, P_DKO*Ex_ < 0.01), which is consistent with the notion that FA uptake by red skeletal muscle and the heart is reduced in DKO mice [3]. Serum levels of glycerol were elevated by exercise (Fig. 2d), suggesting that lipolysis occurred similarly in both WT and DKO mice. Levels of the β-hydroxybutyrate (BHB, ketone body) in serum were increased by DKO genotype and fasting (Fig. 2e). Interaction (Fig. 2e, P_DKO*fast_ < 0.01 and P_DKO*fast*Ex_ < 0.05) further suggests that compensatory utilization of the ketone body was more enhanced during exercise in fasted DKO mice compared to fasted WT mice (Fig. 2e, groups 6 and 8). Levels of lactate in serum were increased by exercise and reduced by fasting (Fig. 2f), suggesting occurrence of anaerobic glycolysis with exercise. As glucose is likely to be an important energy substrate for DKO mice even in the fasted state [3], our findings imply that marked hypoglycaemia due to fasting and FABP4/5 deficiency (Fig. 2a, groups 7 and 8) could cause detrimental effects on the exercise performance in fasted DKO mice.

### Accelerated depletion of energy storage in fasted DKO mice

**Fig. 3:**
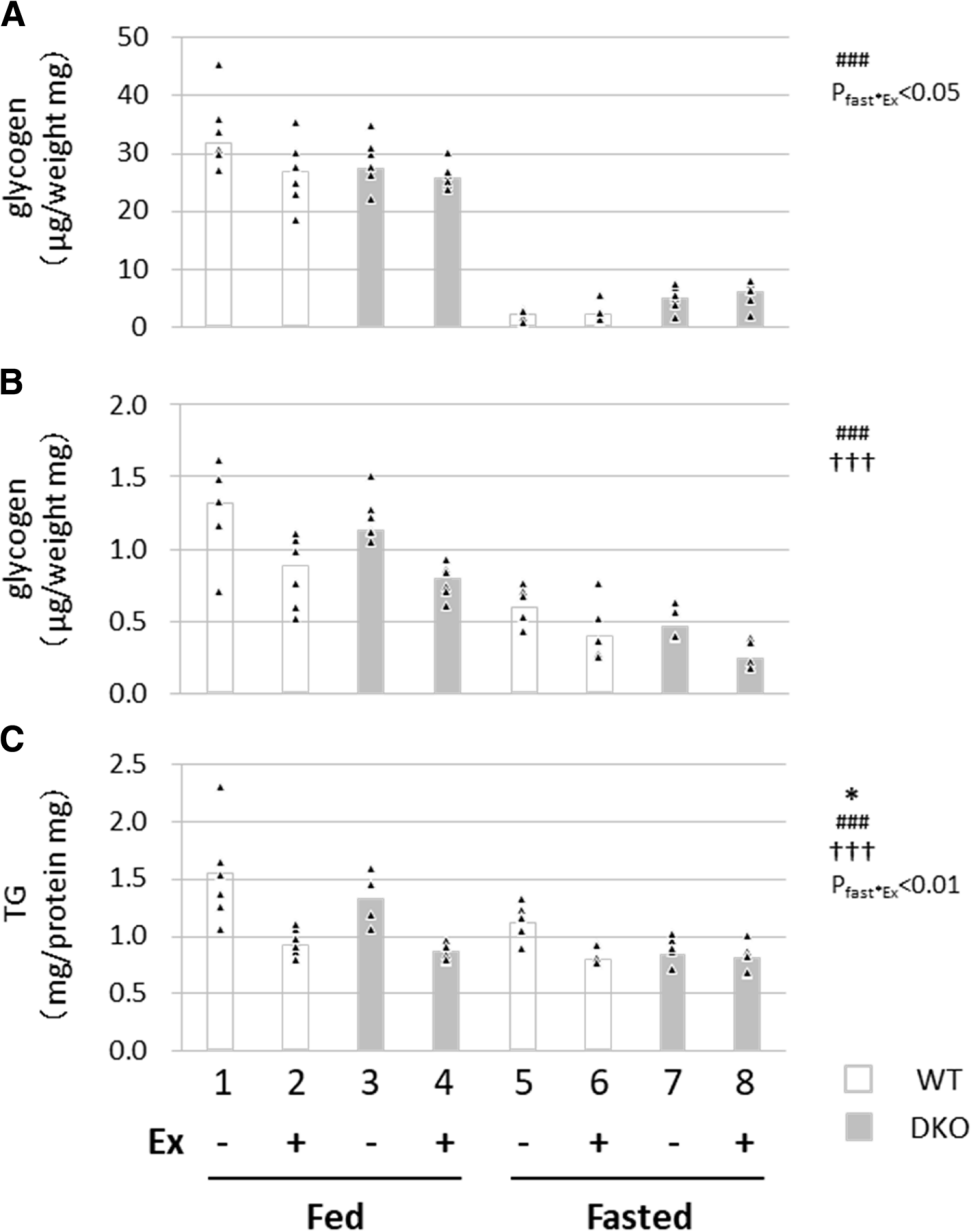
Measurement of energy storage in liver and skeletal muscle. The liver and quadriceps femoris muscle were isolated with or without treadmill exercise in the fed or fasted state to measure glycogen and TG content. (A) Glycogen content in the liver. (B) Glycogen content in the quadriceps femoris. (C) TG content in the quadriceps femoris. TG content was normalized using protein concentration. TG, triacylglycerol. *n* = 6. *p < 0.05, main effect for genotype. ^###^*p* < 0.001, main effect for fasting. ^**†††**^*p* < 0.001, main effect for exercise. Interaction is indicated with the *p*-value in the figure if it was statistically significant

We next examined the storage of glycogen in the liver and glycogen and TG in skeletal muscle before and after exercise. Glycogen levels in the liver were significantly decreased by fasting, leading to the depletion of glycogen in both WT and DKO mice in the fasted state prior to exercise (Fig. 3a). The marginal reduction in glycogen by exercise in the fed state was lost after a 24-h fast (Fig. 3a, P_fast*Ex_ < 0.05), suggesting that glycogen in the liver was unavailable for endurance exercise during prolonged fasting. Levels of glycogen in skeletal muscle were reduced by fasting and exercise (Fig. 3b). Importantly, glycogen levels seemed to be lower in fasted DKO mice after exercise compared to fasted WT mice (Fig. 3b, groups 6 and 8) despite early exhaustion (Fig. 1c), which suggests that muscle glycogen was depleted during an early phase of exercise in DKO mice. TG levels in skeletal muscle were reduced by DKO genotype, fasting and exercise (Fig. 3c). There was no reduction in TG content between pre- and post-exercise in fasted DKO mice (Fig. 3c, groups 7 and 8), suggesting no consumption of intramuscular TG during exercise in fasted DKO mice. Thus, combustion of the energy storage in the liver and skeletal muscle during exercise was markedly diminished during fasting, which is likely to worsen the energy status in DKO mice more severely.

### Expression of genes for gluconeogenesis and ketogenesis in liver was comparable between WT and DKO mice in the fasted state

**Fig. 4:**
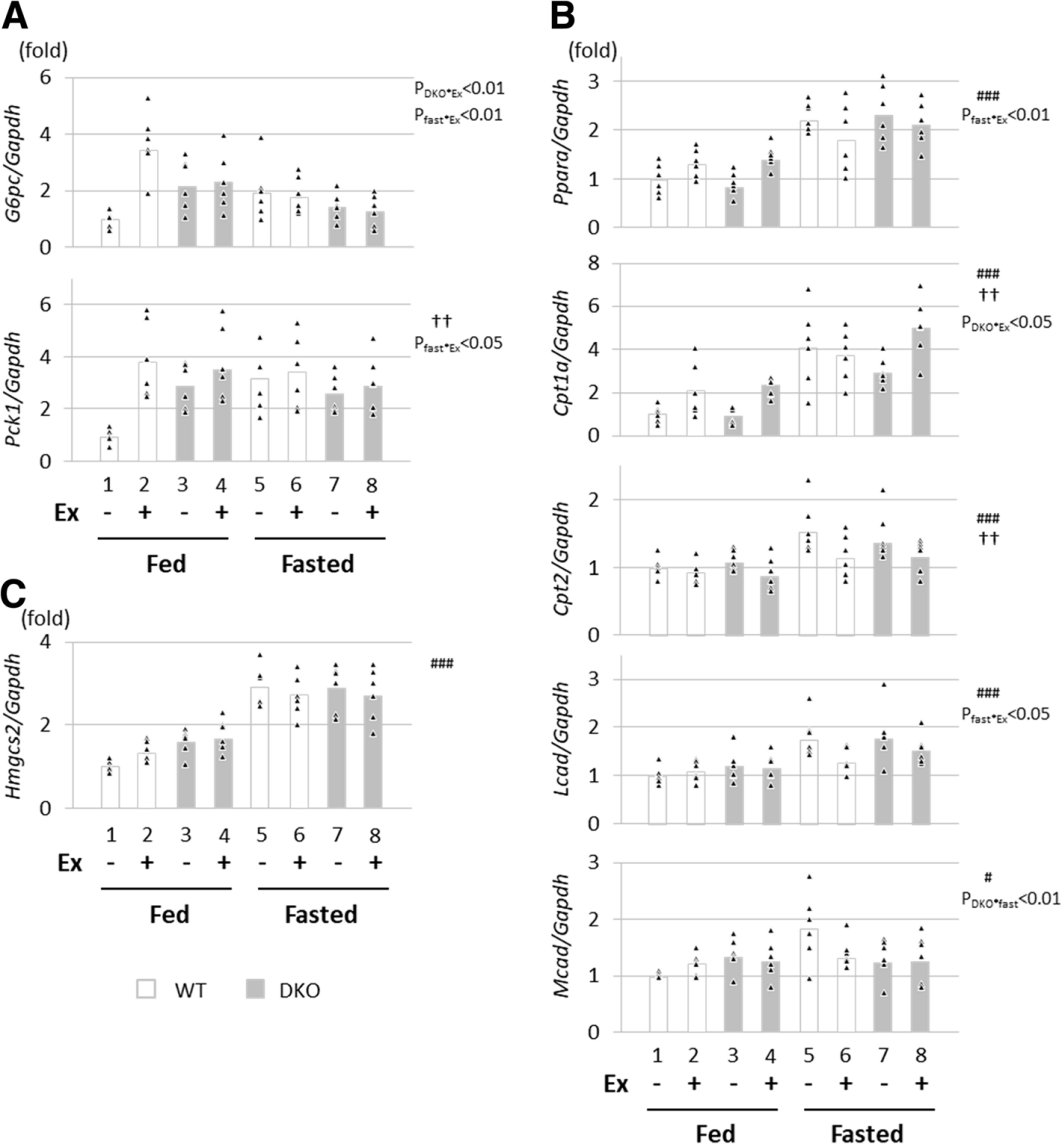
Expression of genes associated with gluconeogenesis, β-oxidation and ketogenesis in liver. The liver was isolated with or without treadmill exercise in the fed or fasted state. The total RNA was extracted for quantitative real-time PCR. (A) Genes associated with gluconeogenesis. (B) Genes associated with β-oxidation. (C) Genes associated with ketogenesis. *G6pc*, glucose-6-phosphate catalytic subunit; *Pck1*, phosphoenolpyruvate carboxykinase; *Hmgcs2,* 3-hydroxy-3-methylglutaryl-CoA synthase 2; *Ppara*, peroxisome proliferator activated receptor α; *Cpt1a/2*, carnitine palmitoyltransferase 1a/2; *Mcad*, medium-chain acyl-CoA dehydrogenase; *Lcad*, long-chain acyl-CoA dehydrogenase. *n* = 6. ^#^*p* < 0.05, ^###^*p* < 0.001, main effect for fasting. ^**††**^*p* < 0.01, main effect for exercise. Interaction is indicated with the p-value in the figure if it was statistically significant

Our previous report demonstrated that gluconeogenesis is markedly reduced in fasted DKO mice as shown by the pyruvate challenge test [16]. In this study, we next studied the mRNA expression of genes regulating gluconeogenesis with and without exercise in the fed and fasted states [15]. The basal expression of the glucose-6-phosphate catalytic subunit (*G6pc*) and phosphoenolpyruvate carboxykinase (*Pck1*) tended to be elevated in DKO mice (Fig. 4a, groups 1 and 3), but there were no significant main effects on their induction for DKO genotype by three-way ANOVA. Although exercise induced the expression of *G6pc* and *Pck1* in fed WT mice, prolonged fasting blunted the effect of gene induction by exercise (Fig. 4a, P_fast*Ex_ < 0.01 for *G6pc*, P_fast*Ex_ < 0.05 for *Pck1*). These findings suggest that compensatory gluconeogenesis was not enhanced in fasted DKO mice, which could contribute to more hypoglycaemia.

We next studied the mRNA expression levels of genes related to β-oxidation and ketogenesis, which were regulated by PPARA [23, 24]. The expression of *Ppara* and β-oxidation related genes (*Cpt1a/2*, *Lcad* and *Mcad*) was induced by fasting and partially affected by exercise, but not influenced by DKO genotype (Fig. 4b), suggesting that β-oxidation activity was not affected in DKO mice when compared to WT mice. The expression of *Hmgcs2*, a rate-limiting enzyme in ketogenesis, was increased by fasting in both WT and DKO mice (Fig. 4c). Thus, there was no enhancement of mRNA expression for gluconeogenesis or ketogenesis in the livers of fasted DKO mice during exercise, which may cause early exhaustion due to no compensatory supply of alternative energy substrates from the liver to the skeletal muscle.

### Expression of genes for energy combustion in skeletal muscle was not reduced in fasted DKO mice

**Fig. 5:**
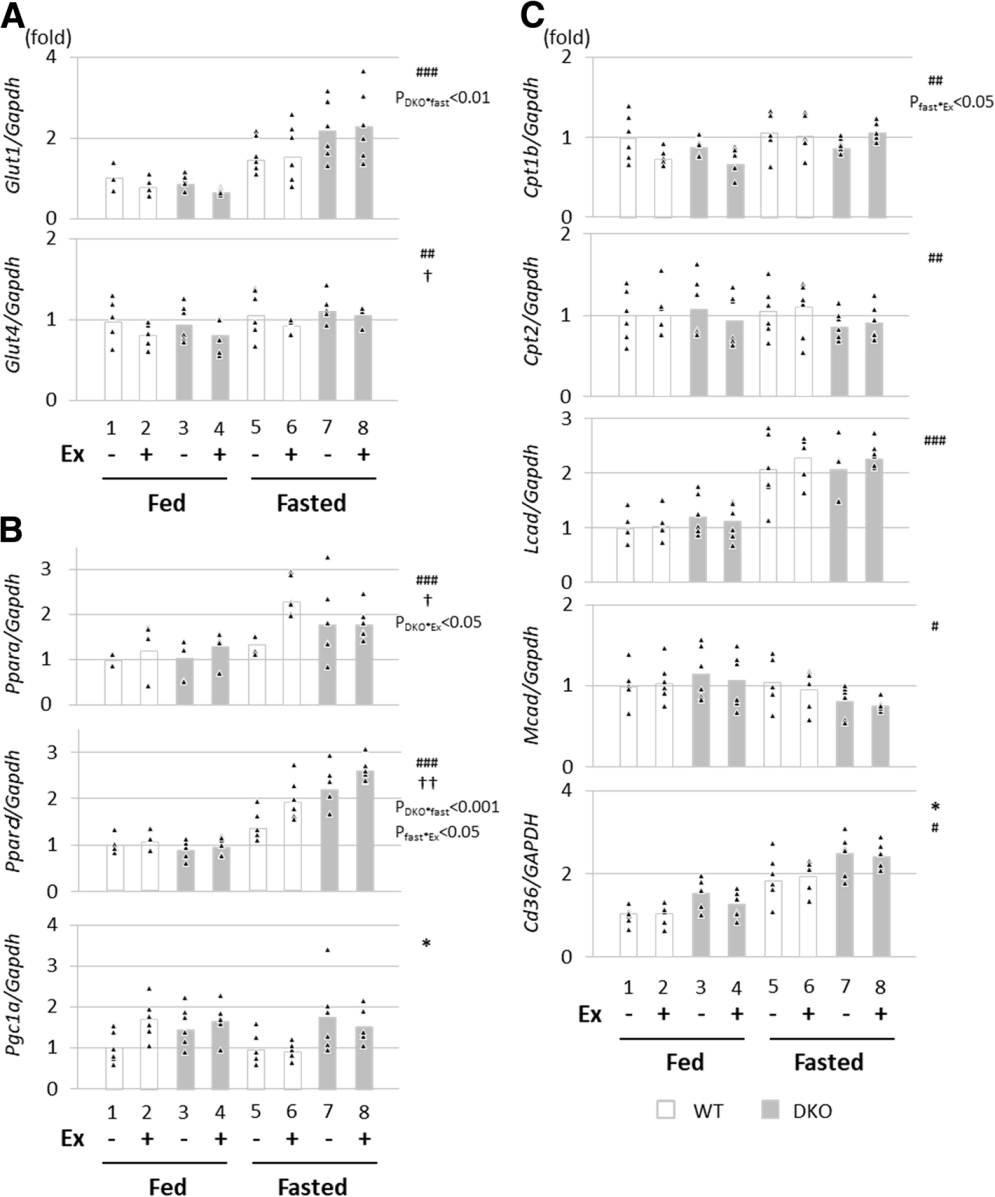
Expression of genes associated with energy metabolism in skeletal muscle. The quadriceps femoris muscle was isolated with or without treadmill exercise in the fed or fasted state. The total RNA was extracted for quantitative real-time PCR. (A) Genes associated with glucose uptake. (B) Genes of central regulators for FA metabolism. (C) Genes associated with β-oxidation and FA uptake. Glut1/4, glucose transporter 1/4; *Ppara*, peroxisome proliferator activated receptor α; *Ppard*, Ppar δ, *Pgc1a*, PPARG coactivator 1α. n = 6. *p < 0.05, main effect for genotype. ^#^*p* < 0.05, ^##^*p* < 0.01, ^###^*p* < 0.001, main effect for fasting, ^**†**^*p* < 0.05, ^**††**^*p* < 0.01, main effect for exercise. Interaction is indicated with the p-value in the figure if it was statistically significant

Next, we studied expression levels of genes associated with glucose and FA metabolism in skeletal muscle. The expression of glucose transporters *Glut1* and *Glut4* was induced by fasting, but not affected by DKO genotype (Fig. 5a). The induction of *Glut1* by fasting was augmented in DKO mice (Fig. 5a, P_DKO*fast_ < 0.01). *Ppara*, *Ppard* and their co-activator, PPARγ-coactivator-1 alpha (*Pgc1a*) are essential transcription factors controling FA combustion [19]. The expression of mRNA for *Ppara* and *Ppard* was induced by fasting and exercise but was not different between WT and DKO mice (Fig. 5b). The expression of *Pgc1a* was marginally elevated by DKO genotype (Fig. 5b). Consist with these data, the expression of target genes of the PPAR/PGC-1 complex was not significantly influenced by DKO genotype, except *Cd36* (Fig. 5c). Thus, there is only a minor difference in terms of the expression of genes associated with glucose and FA metabolism in skeletal muscle, which is unlikely to compensate for reduced energy supply during exercise in fasted DKO mice.

### Composition of fibre types in oxidative skeletal muscle was comparable

**Fig. 6:**
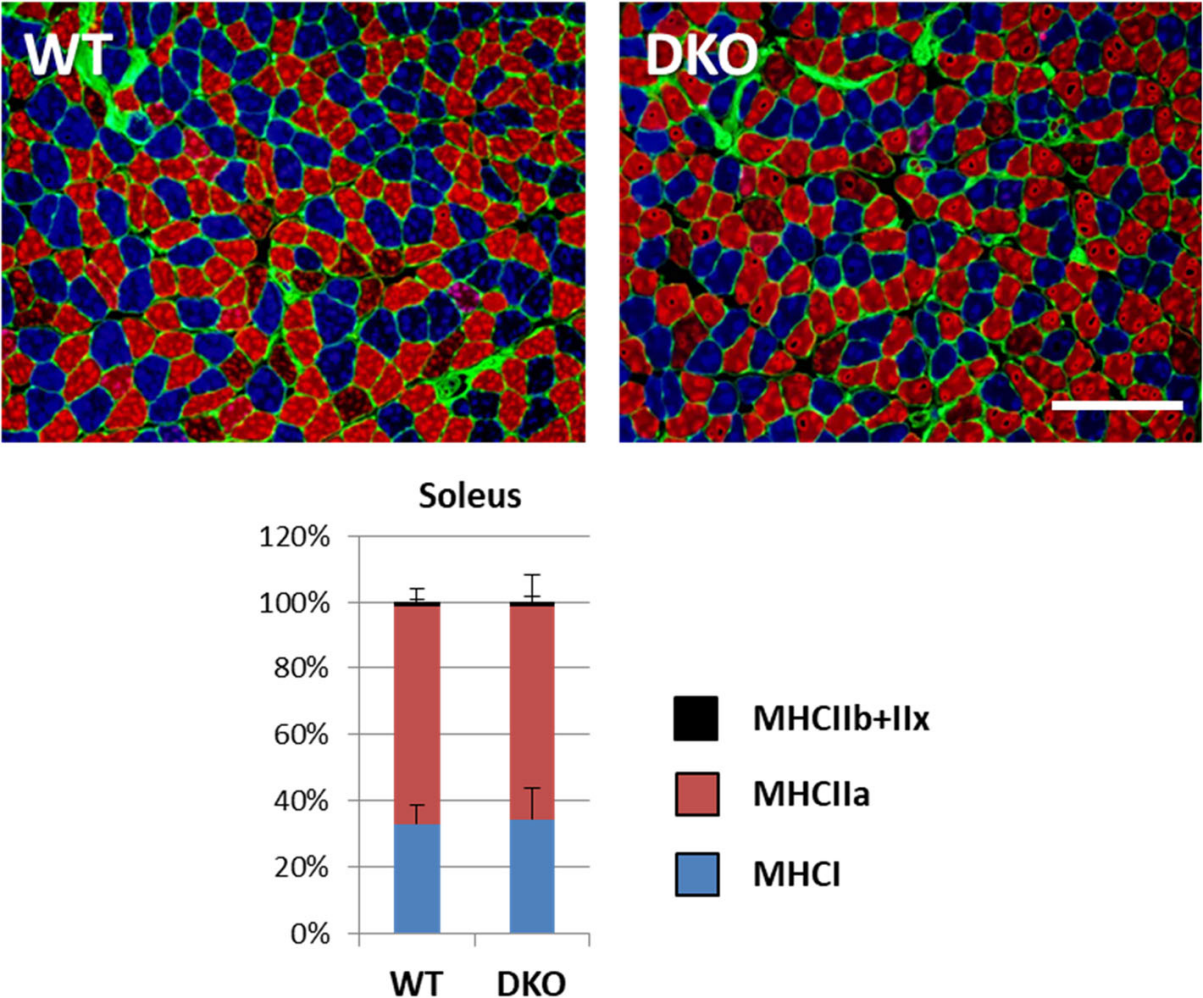
Expression of MHC isoforms in skeletal muscle. The soleus muscle was isolated in WT and DKO mice to estimate the expression of MHC isoforms by immunofluorescence. MHC I and MHC IIa were stained with blue and red, respectively. Muscle fibres negative for MHC I and MHC IIa were represented as a combination of type IIb and IIx fibres. The bar graph shows the percentage of each MHC isoform. MHC, myosin heavy chain. *n* = 6. Scale bar, 100 μm

Oxidative red muscle fibres, which mainly utilize FA, are fatigue-resistant whereas glycolytic white fibres, which rely on more glucose, are susceptible to fatigue [19, 25]. As red/oxidative muscle consumes more glucose in DKO mice, we questioned whether a shift of energy substrates from FA to glucose affects muscle fibre types, which might, in turn, cause an early exhaustion upon exercise during fasting. To determine muscle fibre types in oxidative muscle (i.e., the soleus), immunofluorescence was performed to detect myosin heavy chain (MHC) isoforms (type I, oxidative fibre; type IIb and IIx, glycolytic fibres; type IIb, characteristics between oxidative and glycolytic fibres) [22]. As shown in Fig. 6, there was no significant difference regarding the composition of MHC isoforms. Thus, our data suggest that a reduction in exercise endurance is not caused by the phenotypic change of muscle fibre types.

## Discussion

We demonstrated that FABP4/5 have a critical role in exercise endurance in the fasted state. Energy storage, such as glycogen in the liver and glycogen and TG in skeletal muscle, were almost depleted in fasted DKO mice. Accelerated utilization of glucose in oxidative muscle and the heart is maintained even during fasting and gluconeogenesis is diminished [3], which could result in accelerated hypoglycaemia. Further, FA uptake is decreased in oxidative muscle due to defective trans-endothelial FA transport via capillary endothelial FABP4/5 [3, 16]. As a result, energy supply from serum and energy storage in skeletal muscle are very low in fasted DKO mice prior to exercise, which could bring about a significant reduction in exercise performance. We conclude that deranged uptake and redistribution of energy substrates during prolonged fasting provoke maladaptive energy homeostasis, leading to a significant reduction in exercise endurance capacity.

It is noteworthy that exercise performance was not reduced much in DKO mice compared to WT mice in the fed state (Fig. 1c) when glycogen and TG storage was fulfilled (Fig. 3b and c). We recently reported similar findings in CD36 knockout (KO) mice [20]. Like FABP4/5 DKO mice, CD36KO mice also display a reduction in FA transport and oxidation with a compensatory use of glucose [26, 27]. In CD36KO mice, endurance capacity is severely reduced in the fasted state, while the reduction is modest in the fed state [20]. These findings further support the notion that intact FA utilization by peripheral tissues plays an important role in nutrient homeostasis in the fasted state to maintain the exercise endurance capacity. In contrast to our observation, however, previous studies showed a reduction in exercise endurance capacity in CD36KO mice even during fasting [28–30]. However, between our studies and previous reports with CD36KO mice, there is a major difference regarding the exercise protocols. The treadmill speed used in previous studies was 17 m/min or less (mild to moderate intensity) [28–30]. In our studies, the exercise speed was increased up to 30 m/min in a single bout (high intensity). High-intensity exercise induces maximum reliance on glycogen in skeletal muscle, while mild to moderate intensity exercise leads to modest reliance on intramuscular glycogen and TG, as well as circulating NEFA [31]. In addition to energy reliance, mild to moderate intensity exercise results in a longer exercise time (hours), which could, in turn, augment the relative contribution of FABP4/5/CD36-mediated FA uptake from the circulation. Thus, exercise intensity, as well as duration, affects the preference for main energy substrates for endurance, which can cause differences in the endurance capacity between WT mice and mice with defective FA utilization. In the fed state, endurance capacity for high intensity exercise is more affected by glycogen content in skeletal muscle, whereas the capacity for mild to moderate intensity exercise is more influenced by the FA uptake ability.

In addition to an enhanced reduction in endurance capacity, we reported that DKO mice exhibit lethal hypothermia in response to cold exposure in the fasted state [17]. Accelerated hypothermia during fasting is mainly caused by a marked reduction in TG storage in brown adipose tissue, glycogen in skeletal muscle and blood glucose. Importantly, rapid hypothermia does not occur in the fed state, when energy storage in brown adipose tissue and skeletal muscle is filled. Thus, an inappropriate redistribution of energy substrates occurs in DKO mice only in the fasted state, which leads to the depletion of tissue energy storage for thermogenesis and exercise. Similar responses during fasting were also observed in CD36KO mice, another model with defective FA utilization [20, 32], suggesting that such physiological disadvantages during fasting seem to be a common feature in animals with defective FA utilization. These findings further provide an important notion that in general, appropriate FA utilization by peripheral tissues is essential to adapt to severe natural environments such as famine and coldness, and to chase animals or escape from natural enemies during fasting.

There is accumulating evidence that the gut microbiota plays a role in the host metabolic status as a mediator of dietary intake [33, 34]. Germ-free mice have reduced adiposity, improved tolerance to glucose and insulin and are protected from diet-induced obesity when fed a Western diet [33, 34]. It has also been suggested that an altered composition of the microbiota is associated with increased host adiposity via a number of different mechanisms [33, 34]. In addition, the mouse microbiota is known to be heavily affected by their environment. In this study, control WT mice were purchased from an outside vendor one week before treadmill exercise training, while DKO mice were born and grown in our animal facility. Thus, the difference of housing conditions between WT and DKO mice might affect the results of metabolic data to a certain degree, which needs careful interpretation.

In our previous studies, we estimated cardiac function in DKO mice in pressure overload-induced and lipopolysaccharide-induced cardiomyopathy models [35, 36]. Cardiac dysfunction was more obvious in DKO mice compared to WT mice in both models, presumably due to energy depletion. Interestingly, however, induction levels of mRNA for natriuretic peptide b (*Nppb*), a marker of cardiac stress, were comparable in WT and DKO mice, suggesting that cardiac dysfunction occurs by energy depletion with no change of *Nppb* expression levels (unpublished observation). In this study, we also found comparable induction of *Nppb* in WT and DKO mice after exercise in the fasted state. Based on our previous observation, the result does not necessarily mean that cardiac function is similar between WT and DKO mice. Thus, the possibility that a reduction in exercise endurance capacity in DKO is caused by cardiac dysfunction remains unsolved.

## Conclusions

FA uptake by peripheral tissues via capillary endothelial FABP4/5 has crucial roles in nutrient homeostasis for maintaining exercise endurance capacity during prolonged fasting.

## Abbreviations

ATP: Adenosine triphosphate
BHB: β-hydroxybutyrate
Cpt1b/2: Carnitine palmitoyltransferase 1b/2
DKO: Double knockout
FA: Fatty acid
FABP: Fatty acid binding protein
G6pc: Glucose-6-phosphate catalytic subunit
Gapdh: Glyceraldehyde 3-phosphate dehydrogenase
Glut1/4: Glucose transporter 1/4
Hmgcs2: 3-hydroxy-3-methylglutaryl-CoA synthase 2
Lcad: Long-chain acyl-CoA dehydrogenase
Mcad: Medium-chain acyl-CoA dehydrogenase
MHC: Myosin heavy chain
NEFA: Non-esterified fatty acid
Pck1: Phosphoenolpyruvate carboxykinase
Pgc1a: Peroxisome proliferator activated receptor γ coactivator 1 alpha
Ppara: Peroxisome proliferator activated receptor α
Ppard: Peroxisome proliferator activated receptor δ
TG: Triacylglycerol
WT: Wild type
